# The Incidence of Fracture-Related Infection in Open Tibia Fracture with Different Time Interval of Initial Debridement

**DOI:** 10.5704/MOJ.2211.005

**Published:** 2022-11

**Authors:** D Hadizie, YS Kor, SA Ghani, MA Mohamed-Saat

**Affiliations:** Department of Orthopaedics, Universiti Sains Malaysia, Kubang Kerian, Malaysia

**Keywords:** fracture, tibia, gustilo-anderson, fracture-related infection

## Abstract

**Introduction:**

The primary aim of open fracture management is to prevent fracture-related infection by early antibiotic administration, debridement and wound coverage. However, the timing of the initial debridement is still controversial, and 6 to 24 hours is commonly advocated. Studies have yet to provide substantial evidence regarding the best time for surgical debridement. Thus, this study was conducted to compare the incidence of fracture-related infection at different time intervals of initial debridement of the open tibia fracture.

**Materials and methods:**

A total of 91 patients with grade I, II and IIIa open tibia fractures were recruited from 2016 to 2018, and their data were obtained from the consensus book and medical records. Participants were divided into four groups based on the time of initial debridement: (1) less than 6 hours, (2) 6 to less than 12 hours, (3) 12 to less than 24 hours, and (4) 24 hours and more. Fracture-related infection was determined by using Metsemakers confirmative criteria. Association between time and infection were determine by Binary Logistic Regression analysis by remerged the group into three; (1) less than 12 hours, (2) 12 to less than 24 hours and (3) 24 hours and more. The collected information was analysed using SPSS version 24 and Microsoft Excel 2010.

**Results:**

The mean age of the participants was 31.9 years old, with male predominant (n=80, 87.0%). Most participants had delayed initial debridement of more than 24 hours and predominantly Gustilo-Anderson type IIIa (n=47). A total of 8 fractures complicated with infection (8.7%), majority in grade IIIa and debridement performed within 12 to less than 24 hours. Binary logistic regression showed increased odds of infection with a delayed wound debridement both in clinical presentation and positive culture, but the association was not statistically significant. The commonest organism isolated was Pseudomonas aeruginosa.

**Conclusion:**

Comparing to different time interval, initial wound debridement of more than 24 hours did not have strong association with increasing infection rate. However, even though statistically not significant, the odds of infection was increase with increasing time of initial wound debridement of an open tibia fracture, thus it should be performed early.

## Introduction

An open fracture is defined as a breach of bone continuity associated with soft tissue damage in which it is communicating with the external environment^1^. It is considered an orthopaedic emergency, and its management remains a challenge for orthopaedic surgeons. The primary aim in managing an open fracture is to prevent fracture-related infection, which can be contributed by several factors. Early antibiotic administration, early and aggressive debridement and early wound coverage have drastically reduced the rate of infection in recent years^2^. However, the urgency of debridement has not been clearly defined. Most surgeons advocated for initial debridement within the first six hours, but the classic six-hour rule remains controversial. Meanwhile, some surgeons favour initial debridement within 6 to 24 hours to reduce the incidence of infection and non-union in an open fracture. Nevertheless, there is a lack of substantial evidence regarding the best time for surgical debridement, and no consensus has yet to be achieved. In addition, no study has yet to compare the infection rate based on the specific time interval of initial debridement. Therefore, this study aimed to compare the infection rate at different time intervals of initial debridement of an open fracture.

To date, the definition of infection in open fracture remains controversial. Research findings have yet to determine any criteria to confirm infection in an open fracture, most of them depending on clinical and radiological findings to ascertain infection. Recently, Metsemakers *et al* have reported confirmatory and suggestive criteria to diagnose fracture-related infection^3^. These criteria that have not been previously tested were used in the present study to diagnose open fracture-related infection.

## Materials and Methods

This study involved 91 patients admitted to our centre from 2016 to 2018. Patients’ data were traced and collected from the consensus book in the operation theatre and included in the study if they fit the inclusion and exclusion criteria. All patients, regardless of race and ages, sustained open tibia fracture Gustilo-Anderson grade I, II and IIIa were included in this study. On the other hand, patients with polytrauma, mental illness, immunocompromising diseases such as diabetes, malignancy or steroid usage and open fracture tibia grade IIIb and IIIc were excluded from the study.

Additional information required for the study were traced from the medical record unit. Information related to the study were collected on the designated datasheets and analysed using SPSS version 24 and Microsoft Excel 2010. The patients were divided into four groups based on time of initial wound debridement: (1) less than 6 hours, (2) six to less than 12 hours, (3) 12 to less than 24 hours, and (4) 24 hours and more.

Sociodemographic and clinical characteristics of all patients included were described as mean with its standard deviation (SD) for numerical variable, and frequency (n) and its percentage (%) for categorical variables.

Fracture-related infection is determined by using Metsemakers *et al* confirmatory criteria according to two criterias: (1) presence of fistula, sinus, wound breakdown, purulent discharge, or presence of pus, and (2) positive culture (deep tissue sample) obtained intra-operatively and confirmed by histopathological examination using specific staining techniques or bacteria or fungi^3^. If the patients’ condition fulfils the confirmatory criteria, fracture-related infection was determined.

Furthermore, the time of debridement for each Gustilo-Anderson fracture grade and the number of infections based on time of initial debridement were described as n and %. The association between time of initial debridement and infection was determined by using Binary Logistic Regression analysis. The time for initial debridement was merged into either; (1) less than 12 hours, (2) between 12 and less than 24 hours and (3) 24 hours and more. This is because no infection was observed among those who underwent debridement between 6 and less than 12 hours. The odds of infection were compared to those who underwent debridement of less than 12 hours. The results were presented as odds ratio (OR), 95% confidence interval (95% CI) and p-value. A p-value of less than 0.05 was considered statistically significant. This study has been approved by the Human Research Ethics Committee.

## Results

Total of 91 patients involved in this study. Majority of them were males (n=79, 86.8%), and 12 patients were females (13.2%). Besides, most participants were Malay (n=90, 98.9%) and only one patient was Chinese (n=1, 1.1%). The patients’ age in this study ranged from 6 to 71 years, with a mean age of 31.9±17.7. Based on the Gustilo-Anderson open tibia fracture grading, 46 patients sustained grade IIIa fractures (50.5%), followed by grade II (n=30, 33.0%) and grade I (n=15, 16.5%). The socio-demographic characteristics of the participants in this study are summarised in Table I.

**Table I:** Sociodemographic and clinical characteristics of patients (n=91)

Variable	Mean (SD) / n (%)
Age	31.85 (17.81)
Sex	
Female	12 (13.2)
Male	79 (86.8)
Race	
Malay	90 (98.9)
Chinese	1 (1.1)
Gustilo-Anderson grade	
Grade I	15 (16.5)
Grade II	30 (33.0)
Grade IIIa	46 (50.5)
Duration of initial trauma to debridement(hour)	
< 6h	9 (9.9)
Between 6h and <12h	21 (23.1)
Between 12h and < 24h	28 (30.8)
≥ 24h	33 (36.3)
Fistula, sinus, wound breakdown, purulent discharge or presence of pus	
No	83 (91.2)
Yes	8 (8.8)
Positive culture	
No	85 (93.4)
Yes	6 (6.6)

Note; Age range: min = 6, max = 71

Based on the findings, most patients with open tibia fractures experienced delayed initial debridement of more than 24 hours (n=33, 36.3%), followed by 12 to less than 24 hours (n=28, 30.8%), 6 to less than 12 hours (n=21, 23.1%) and less than 6 hours (n=9, 9.9%) group. Majority of initial debridement among patients with grade I and grade II were performed at more than 24 hours (n=6, 0.0% and n=13, 43.3%). Meanwhile, among patients with grade IIIa fracture, majority of debridement was performed between 12- and 24-hours post trauma (n=16, 34.8%).

The total number of infections based on wound breakdown, pus and sinus was 8.7%, consisting of 8 out of 91 patients. Whereas the total fracture-related infection rate based on positive organisms was 6.6%, consisting of 6 out of 91 patients. Furthermore, fracture-related infection was most common in those with debridement performed less than 6 hours (11.1%), followed by those with debridement performed at and more than 24 hours (9.1%), and between 12 and less than 24 hours (7.1%). There was no infection detected among those with debridement performed between 6 and less than 12 hours. These findings are summarised in Table II and III.

**Table II:** Gustilo-Anderson grade and time of debridement

Gustilo-Anderson grade		Time of debridement, n (%)		
	< 6h	Between 6h and <	12h Between 12h and < 24h	≥ 24h
Grade I	1 (6.7)	3 (20.0)	5 (33.3)	6 (40.0)
Grade II	2 (6.7)	8 (26.7)	7 (23.3)	13 (43.3)
Grade IIIa	6 (13.0)	10 (21.7)	16 (34.8)	14 (30.4)

**Table III:** Rate of infection per time group

Duration of initial trauma to debridement (hour)	Total fracture, n	Number of infections based on wound breakdown, pus, and sinus, n (%)	Number of infections based on positive culture, n (%)
< 6h	9	1 (11.1)	1 (11.1)
Between 6h and < 12h	21	0 (0.0)	0 (0.0)
Between 12h and < 24h	28	4 (14.3)	2 (7.1)
≥ 24h	33	3 (9.1)	3 (9.1)

Binary logistic regression analysis was used to examine the association between time intervals of initial debridement with the two definitive fracture-related infection criteria (status of wound breakdown, pus and sinus and positive microorganism isolated). This study found that eight fracture-related infection cases had fulfilled confirmatory criteria (pus discharge, presence of wound breakdown and sinus), whereas six of them resulted in a positive culture of microorganism intra-operatively.

The time interval was recategorised into less than 12 hours, 12 to less than 24 hours, and 24 hours and more because of the small sample size and no infection occurred within time interval of 6 and less than 12 hours.

For the association between time intervals of initial debridement and infection based on fracture-related infection criteria (status of wound breakdown, pus and sinus), patients who underwent debridement at 12 to less than 24 hours had higher odds of infection (OR=4.83) compared to those who underwent debridement of less than 12 hours. Similarly, patients who underwent debridement at 24 hours and more had higher odds of infection (OR=2.90 compared to those who underwent debridement of less than 12 hours. These associations, however, was not statistically significant (Table IV).

**Table IV:** Binary logistic regression analysis to determine association between time to debridement and infection (based on wound breakdown, pus and sinus)

Duration of initial trauma to debridement (hour)	Crude b	Crude OR (95% CI)	p-value
< 12h	Ref		
≥ 12h to < 24h	1.58	4.83 (0.51, 46.18)	0.171
≥ 24h	1.07	2.90 (0.29, 29.51)	0.368

Based on positive microorganism isolated, patients who underwent debridement at 12 hours to less than 24 hours had higher odds of infection (OR=2.23) compared to those underwent debridement of less than 12 hours. Patients who underwent debridement at 24 hours and more had higher odds of infection (OR=2.90) compared to those who underwent debridement of less than 12 hours. Similarly, these association, was not statistically significant (Table V). Table VI summarised the fracture-related infection in patients with their features of infection and positive bacteria culture. The most common bacterial species identified were Pseudomonas aeruginosa, followed by Staphylococcus aureus, Enterobacter species and coagulase-negative Staphylococcus. A total of four patients with fracture-related infection has two or more bacteria cultured from different types of cultures such as pus and tissues.

**Table V:** Binary logistic regression analysis to determine association between time to debridement and infection (based on positive micro-organism isolated)

Duration of initial trauma to debridement (hour)	Crude b	Crude OR (95% CI)	p-value
< 12h	Ref		
≥ 12h to < 24h	0.80	2.23 (1.91, 26.06)	0.522
≥ 24h	1.07	2.90 (0.29, 29.51)	0.368

**Table VI:** Types of organisms obtained from culture and sensitivity

Types of bacteria	n (%)
Pseudomonas aeruginosa	4 (44.4)
Coagulase-negative staphylococcus	1 (11.1)
Staphylococcus aureus	2 (22.2)
Enterobacter species	2 (22.2)

## Discussion

The key principles in open fracture treatment are appropriate antibiotics, debridement, skeletal stabilisation and early soft tissue cover^2^. However, the critical timing of initial debridement remains controversial. The earliest animal study conducted by Friedrich *et al* 1898 showed that the surgical debridement done before six hours drastically reduced the infection eradication measures due to the exponential increase in bacterial replication^4^. This finding was supported by Robson *et al* in who demonstrated that debridement carried out after 6 hours increased the infection rate in human wound^5^. Furthermore, Kreder and Amstrong found that the infection rate in patients who underwent debridement after more than 6 hours was 25% compared to 12% in those treated below 6 hours^6^. After the introduction of Gustilo-Anderson grading for open fracture^1^, Kindsfater and Jonassen utilised this system to correlate the timing of surgery with infection rate and discovered that there was a significant increase in infection rate in grade II and III open fractures that were debrided after five hours. Positive bone cultures were used as confirmatory criteria instead of clinical signs of infection to determine infection status^7^. In 2007, Owen and Wenke did a histological study on biofilm formation in open fractures and revealed that immature biofilm formation began at 5.17 hours of exposure and became matured within 10 hours^8^.

Despite earlier discoveries, recent studies reported no statistical significance between the timing of debridement and the incidence of infection. In 1989, Patzakis and Wilkin studied 1104 open fractures and found no significant effect between the timing of debridement (greater or less than 12 hours of initial debridement) and infection rate. Moreover, it was concluded that the most important predictor of infection rate was the initial administration of broad-spectrum antibiotic less than three hours post-injury^9^. In the present study, the most common antibiotics provided to patients were IV cefuroxime 750mg three times daily, IV metronidazole 500mg three times daily, and IV gentamycin 160mg daily depending on the grade of open fractures. Meanwhile, a recent study by Reuss and Cole reported the lack of relationship between debridement carried out within 6 hours up to 48 hours and deep infection. It was also noted that patients who underwent multiple debridements tend to have a higher infection rate^10^. On top of that, Crowley *et al* concluded that the ‘six-hour rule’ in surgical debridement in open fractures should be re-evaluated and suggested that surgery should be done at the earliest opportunity by experienced orthopaedic and plastic surgeons^11^.

Currently, there is a controversy in defining infection in open fractures. Most studies quoted deep infection based on their clinical findings, and some considered histology findings to determine clinical infection^12^. Fortunately, in 2017, Metsermakers *et al* concluded the recent definition of fracture-related infection achieved through the consensus of a group of international experts. The confirmatory criteria defined by the Metsermakers *et al* were used in the present study to determine the clinical infection in open tibia fractures^3^. Nonetheless, there is a drawback with the criteria due to the absence of a scoring system to determine fracture-related infection objectively.

Several time intervals of initial debridement were correlated with fracture-related infection rates in this study, but no statistical significance was found, as proven by earlier studies^4,5,9,12^. In addition, this study excluded open fracture Gustilo-Anderson grade IIIb and IIIc, which showed a greater infection rate in the study by Singh *et al*^13^. Nevertheless, the present study revealed that grade IIIa patients who underwent initial debridement of more than 24 hours did not have a statistically significant increase in fracture-related infection. This finding is very important considering the timing required for the transfer of the patient from district hospital to a tertiary hospital which has experienced surgeons with adequate medical facilities and better patient outcome. This will contribute to delay in immediate access to operating theatres for such injuries^12^. Similarly, delayed debridement for some injuries, because of the long waiting time for the surgery, may not influence the outcome in a busy hospital with high volume of patients.

The total fracture-related infection cases detected in our centre from 2016 to 2018 were 8.7% of total cases (8 cases), comparable to Reuss and Cole at 8.6%^10^. The low infection rate in the present study may be contributed by the exclusion of higher grades open fractures (grade IIIb and IIIc) known for higher infection rates due to severe soft tissue injury, fracture comminution and neurovascular injury^14^. In addition, some studies reported infection rates of approximately 10 to 15%^15-17^.

All of the infected cases, presented with the clinical findings such as wound breakdown, sinus and pus discharge, had a positive culture intra-operatively except for two patients. Majority of them were sustained grade IIIa fracture. Even though the initial debridement was performed in less than 24 hours, and they did not have any comorbidities, infection did occur. The higher percentage was noted in the less than 6 hours group, but the number showed higher incidence in patient with grade IIIa, and debridement done after 24 hours. We unable to explain the cause of infection in a 17-year-old man with grade II fracture, had no comorbidities and debridement was performed at 5 hours of injury. Apart from that, our studies also found that the odds of infection is higher in those who underwent debridement longer than 12 hours post trauma but was not significantly associated.

Thus, based on these findings, the other factors such as severity of the fractures and soft tissue injuries, thoroughness of the debridement, experience of the surgeon, postoperative wound management should be taken into consideration and might influence the outcomes. The strict six-hour immediate debridement for open fractures (grade I, II and IIIa) should be re-evaluated, and proper pre-operative assessment, availability of surgeons and medical facilities should be considered when dealing with these cases. From this study, in open fractures such as grade I, II and IIIa there is no urgency to go for immediate surgery especially at night where no available surgeons and supporting staffs available. The most common organism isolated in our infected cases were *Pseudomonas aeruginosa,* followed by *Staphylococcus aureus,* Enterobacter species and coagulase-negative Staphylococcus. This is corresponded with Guerra *et al* study in which he found that *Staphylococcus aureus* and Enterobacter aerogenes were the two commonest organisms identified. He also reported that 72% of his infected cases were occurred in grade III open fracture^17^. Fracture related infection was identified to be caused mainly by *Staphylococcus aureus* (30-42%) as well in a review by Depypere *et al*^18^. The current consensus stated that only 8% of the organism cultured from the initial wound prior to the debridement responsible for the deep infection based on the study by Lee *et al*^19^. In view of that, initial culture has not been part of the practice in the management of open fracture in our centre. Thus, no comparison can be made with the organism isolated in our series with the initial organism prior to debridement.

The small sample size was a limitation for this study, but the current data can be used as a baseline for future studies with larger sample size, especially in Malaysia or Asian countries. Despite that, some factors that might lead to outcome variability must be considered, such as mode of injuries, different cultural background and microorganism species present in different geographical locations in managing open tibia fracture.

## Conclusion

Comparing to different time interval, initial wound debridement of more than 24 hours did not have strong association with increasing infection rate. However, even though statistically not significant, the odds of infection was increase with increasing time of initial wound debridement of an open tibia fracture, thus it should be performed early. Based on this study, we believe that the time of initial wound debridement is not the only factors that influence the outcome of an open fractures. Severity of the fractures, soft tissue injuries, efficiency of the debridement and comorbidities of the patient are among the other factors that should be carefully taken into consideration.
